# Zosuquidar restores drug sensitivity in P-glycoprotein expressing acute myeloid leukemia (AML)

**DOI:** 10.1186/1471-2407-8-51

**Published:** 2008-02-13

**Authors:** Ruoping Tang, Anne-Marie Faussat, Jean-Yves Perrot, Zora Marjanovic, Simy Cohen, Thomas Storme, Hamid Morjani, Ollivier Legrand, Jean-Pierre Marie

**Affiliations:** 1 INSERM, U872 Equipe 18 Paris, F-75006 France; 2 Centre de Recherche des Cordeliers, Université Pierre et Marie Curie – Paris6, UMR S U872 Paris, F-75006 France; 3 Assistance Publique – Hôpitaux de Paris; Hôpital Hôtel Dieu, 1 place du Parvis de Notre-Dame, 75181 Paris cedex 04, France; 4 JE Onco-Pharmacologie, IFR53; UFR de Pharmacie, 51096 Reims cedex, France

## Abstract

**Background:**

Chemotherapeutic drug efflux via the P-glycoprotein (P-gp) transporter encoded by the MDR1/ABCB1 gene is a significant cause of drug resistance in numerous malignancies, including acute leukemias, especially in older patients with acute myeloid leukemia (AML). Therefore, the P-gp modulators that block P-gp-mediated drug efflux have been developed, and used in combination with standard chemotherapy. In this paper, the capacity of zosuquidar, a specific P-gp modulator, to reverse chemoresistance was examined in both leukemia cell lines and primary AML blasts.

**Methods:**

The transporter protein expressions were analyzed by flow cytometry using their specific antibodies. The protein functionalities were assessed by the uptake of their fluorescence substrates in presence or absence their specific modulators. The drug cytotoxicity was evaluated by MTT test.

**Results:**

Zosuquidar completely or partially restored drug sensitivity in all P-gp-expressing leukemia cell lines tested and enhanced the cytotoxicity of anthracyclines (daunorubicin, idarubicin, mitoxantrone) and gemtuzumab ozogamicin (Mylotarg) in primary AML blasts with active P-gp. In addition, P-gp inhibition by zosuquidar was found to be more potent than cyclosporine A in cells with highly active P-gp.

**Conclusion:**

These *in vitro *studies suggest that zosuquidar may be an effective adjunct to cytotoxic chemotherapy for AML patients whose blasts express P-gp, especially for older patients.

## Background

Outcomes for patients with acute myeloid leukaemia (AML), particularly those over age 60 years, have not significantly improved in the past 20 years and conventional cytarabine and anthracycline-based chemotherapy remains the gold standard. Despite the activity of these agents, 20% of patients ≤ 60 years and 50% of older patients fail to achieve remission with these standard agents, and only a small proportion patients have a prolonged disease-free survival [1]. Chemoresistance to standard agents has been shown to be related, in part, to overexpression of P-gp, one of the best characterized multidrug resistance (MDR) ABC proteins. P-gp functions by pumping certain drugs out of cells through an active, energy dependent mechanism [2-4].

P-gp expression tends to be increased in older patients with AML and likely contributes to their poor response to induction chemotherapy. Therefore, significant interest has developed in combining modulators that block P-gp-mediated drug efflux with standard chemotherapy regimens. However, randomized trials of P-gp modulators such as cyclosporine A (CsA) and PSC-833 in relapsed or refractory AML patients have had variable results [5-7]. One of the challenges of the use of CsA and PSC-833 has been lack of specificity. In addition to P-gp modulation, both drugs also alter the pharmacokinetic profiles and decrease the clearance of co-administrated chemotherapeutic agents. It has been suggested that the decreased clearance results from modulation of several ABC transporters at hepatic level, as well as altered regulation of cytochrome P450 metabolic enzymes such as CYP3A4 or CYP2C8 [8,9]. As a result, the doses of the chemotherapeutic agents that are substrates for P-gp (DNR, mitoxantrone, etoposide) was reduced by 22% to 66% when used in combination trials with CsA and PCS-833 to avoid excessive toxicity [7,10,11]. In contrast, zosuquidar, a highly specific P-gp inhibitor, which does not interact with other transporters including MRP1, MRP2 and mutant BCRP (R482T) [12], has been developed in an attempt to avoid significant pharmacokinetic interactions and therefore allow co-administration of standard dosing of cytotoxic chemotherapy. Zosuquidar has significantly lower affinity for CYP3A than for P-gp [13] and phase I trials have shown that zosuquidar can be given safely to the AML patients in combination with daunorubicin and cytarabine [14,15]. In addition to the conventional cytarabine and anthracycline-based chemotherapy, Mylotarg is a novel immunoconjugate therapy for acute myeloid leukemia (AML). P-glycoprotein (Pgp) has been shown to confer resistance to Mylotarg and is associated with a worse clinical response. In vitro studies have been showed that inhibition of Pgp function by CsA could restore Mylotarg sensitivity [16,17]. Zosuquidar, a Pgp specific inhibitor, can probably also restore Mylotarg sensitivity.

In this study, we investigate the ability of zosuquidar to reverse resistance to several chemotherapeutic agents which are P-gp substrates and used in the AML treatments or AML trials as well as the capacity of zosuquidar to restore drug sensitivity in a panel of myeloid leukemia cell lines with different levels of P-gp activity. Clinically, it will be important to identify AML patients whose blasts possess high P-gp activity, as this subgroup will be most likely to benefit from combination therapy with zosuquidar. Therefore, we studied the correlation between P-gp activity in primary AML patient blasts and in vitro chemosensitization by zosuquidar.

## Methods

### Cell lines

The studies were carried out with human Bcr-abl myeloid leukemia cells (K562), human myeloid leukemia cells (HL60) and six variant cell lines expressing P-gp, MRP1, or BCRP: K562/HHT40, K562/HHT90 (developed in our laboratory) [18], K562/DOX, K562/BCRP (gift from Y. Suquimoto, Foundation for Cancer Research, Japan) [19], HL60/DNR and HL60/ADR. Cells were cultured in RPMI 1640 medium containing 10% fetal calf serum (FCS), penicillin 50 U/ml, and streptomycin 50 μg/ml and incubated in a humidified atmosphere containing 5% CO_2 _at 37°C.

### AML patient samples

Peripheral blood samples from 31 AML patients were obtained after their informed consent. Mononuclear cells (MNC) were isolated using Ficoll-Hypaque density gradient. The primary AML blasts were cultured under the same general conditions as described above for the cell lines.

### Ethical approval

The present study carried out on human blood cells is in compliance with the Helsinki Declaration, and was approved by the French Institute National of Cancer (Tumo06). AML patient blood samples were obtained after their informed consent (Formulary EORTC study N° 06012).

### P-gp, MRP1, MRP3 and BCRP expression analysis

P-gp, MRP1, MRP3 and BCRP expression was studied by using UIC2 (P-gp, Immunotech, France), QCRL3 (MRP1), MRP3 and Bxp21 (BCRP) monoclonal antibodies (all from Alexis, CA, USA), then labeled with a secondary antibody conjugated with phycoerythrin. Cells (1 × 10^6^) were fixed and permeabilized using IntraPrep™ (Beckman Coulter, Villepinte, France) according to the instructions of the manufacturer. The fluorescence was measured and analyzed by flow cytometry. Protein expression for each transporter was quantified as the mean fluorescence intensity (MFI) shift (ratio of the MFI of antibody and isotype control). All experiments were performed in triplicate.

### P-gp, MRP1 and BCRP activity analysis

Cells (1 × 10^6^) were incubated with 25 nM DiOC_2_(3) or 0.2 μg/ml rhodamine at 37°C for 30 min in presence or absence of either 0.3 μM zosuquidar (Kanisa, USA) or 2 μM CsA for P-gp activity analysis, with 0.2 μM Calcein-AM (C-AM)(Molecular probes, USA) in presence or absence of 5 μM MK571 for MRP1 activity analysis, and with 3 μM mitoxantrone (Mitox) in presence or absence of 10 μM Fumitrimorgin C (FTC) (Alexis Biochemicals, USA) for BCRP activity analysis. Cells were immediately analyzed by flow cytometry. Dye uptake was expressed as D value ranging from 0 (no difference) to 1 (no overlap) generated by Kolmogorove-Smirnov test which was used to determine the distribution of the MFI between presence and absence of modulator. For each sample, 5000 events were collected. All experiments were performed in triplicate.

### Cell viability study (MTT assay)

Cells (2 × 10^4 ^cells/well for cell line, 4 × 10^5 ^cells/well for patient cells) were cultured in 96-well plates. Each drug of interest was added at escalating concentrations in the presence or absence of either zosuquidar or CsA. After 48 hour incubation (except Mylotarg, 4 days incubation), 20 μl of MTT (3-(4,5-Dimethyl-2-thiazolyl)-2,5-diphenyl-2H-tetrazolium bromide) was added to each well for a further 4 hour incubation. The purple precipitate was dissolved in 200 μl DMSO, and the optic density (OD) was determined by the multi-well plate reader (Multiskan Ascent, Labsystems). Each condition was repeated in four wells, and result expressed as the mean of the four wells. The viability is expressed as the ratio of the OD of the cells in presence of each drug at different concentration with or without modulator and the OD of control cells in media without drug. The IC_50 _(the half maximal inhibitory concentration) was determined by Software (Biosoft, Cambridge, UK) following the viability results. All experiments were performed in triplicate.

### Statistical analysis

The statistical analysis was performed by the statistical discovery software (JMP5.1) en using student's t-test of comparisons for each pair.

## Results

### Protein expression of P-gp, MRP1, MRP3 and BCRP in K562, HL60 and variant cell lines

**Figure 1:**
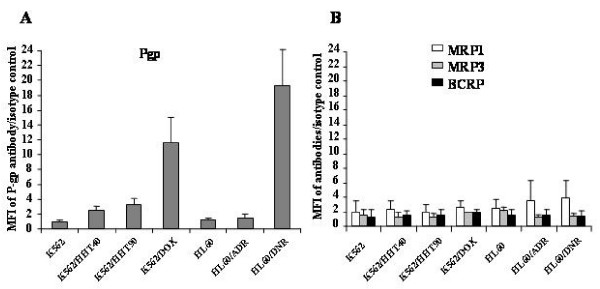
**P-gp, MRP1, MRP3 and BCRP expression in K562, HL60 and their variant resistant cell lines**. A) P-gp expression was analyzed by UIC2 monoclonal antibody. B) MRP1, MRP3 and BCRP were analyzed respectively by their specific monoclonal antibodies QCRL3 (white), MRP3 (grey) and Bxp21 (black).

First, P-gp expression was evaluated in K562, HL60 and variant cell lines. P-gp expression in resistant variant cell lines K562/HHT40, K562/HHT90 and K562/DOX, was increased compared to parental K562S cells (MFI = 0.98 ± 0.17), with MFI shifts of 2.48 ± 0.60, 3.24 ± 0.80, and 11.58 ± 3.42 respectively. HL60/DNR cells expressed strongly P-gp (19.30 ± 4.79), but HL60/ADR cells did not show significant higher P-gp expression (1.50 ± 0.44) than parental HL60/S cells (1.14 ± 0.27) (Figure 1A).

Second, in order to characterize whether there is a cross-resistance in these cell lines, MRP1, MRP3, and BCRP expression was also studied. The expression of MRP1 was similar in these cell lines with a few exceptions. MRP1 expression in HL60/ADR and HL60/DNR was higher than the HL60 parental cell line with MFI shifts of 3.50 ± 2.84 and 3.81 ± 2.44 respectively. No significant MRP3 or BCRP expression was observed in any cell lines tested (Figure 1B).

### P-gp, MRP, and BCRP activity in K562, HL60 and variant cell lines

**Figure 2:**
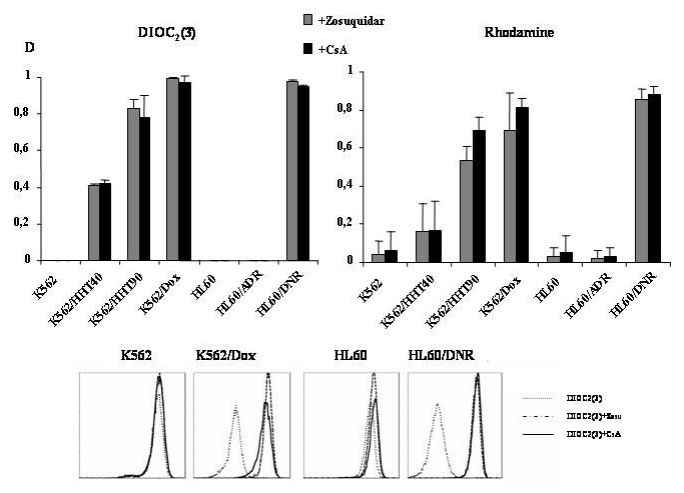
**P-gp activity in K562, HL60 and their variant resistant cell lines**. P-gp activity was measured by the uptake of DiOC_2_(3) and rhodamine in either presence or absence of either zosuquidar (grey) or CsA (black). Two examples histograms K562 versus K562/Dox and HL60 versus HL60/DNR are presented in this figure.

In some cases, the ABC protein expression does not conduct to their capacity to transport their substrate in cells. Therefore, we have examined P-gp, MRP1 and BCRP activity in those cell lines. P-gp activity was assessed by the uptake of two different fluorescent substrates DiOC_2_(3) and rhodamine in the presence or absence of either zosuquidar or CsA. The results are displayed in Figure 2. The values of P-gp activity measured by the uptake of DiOC_2_(3) ± zosuquidar or CsA as modulator were similar to that measured by the uptake of Rhodamine. P-gp activity of K562/HHT40, K562/HHT90 and K562/DOX cells was increased compared to the parental K562 cells. Interestingly, HL60/DNR cells showed very high P-gp activity, while HL60/ADR cells had similar P-gp activity to parental HL60 cells. These P-gp activity results or pump activities correlate closely with P-gp protein expression.

**Figure 3:**
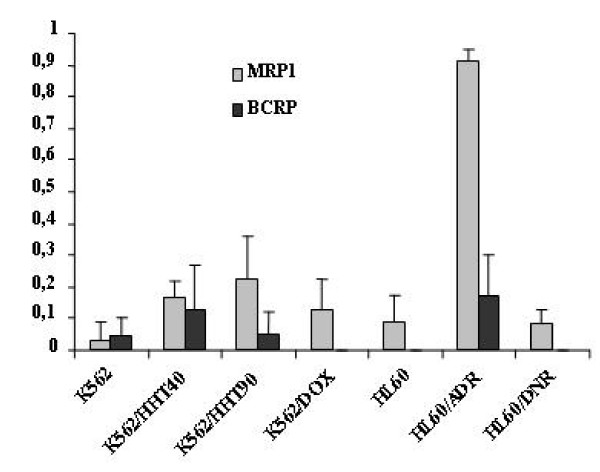
**MRP1 and BCRP activity in K562, HL60 and their variant resistant cell lines**. MRP1 and BCRP activity was measured by the uptake of Calcein-AM and Mitox in presence or absence of their specific modulators MK571 (grey) and FTC (black).

BCRP and MRP activity was analyzed by the uptake of their corresponding substrates (Mitox and C-AM), in the presence or absence of their respective specific modulators (FTC and MK571). Only HL60/ADR cells exhibited significant MRP activity (D = 0.91 ± 0.04) (Figure 3). This correlates with the finding of significant MRP protein expression in this cell line compared to the other cell lines evaluated. But HL60/DNR cells did not exhibit significant MRP activity (0.09 ± 0.04). No cell line demonstrated significant BCRP activity (Figure 3).

### Modulation of drug resistance in K562 and HL60 variant cell lines

The effects of zosuquidar and CsA on the cytotoxicity of several drugs currently used in the treatment of AML or in clinical investigation for AML therapy were examined in these escalating resistant cell lines. The chemotherapy agents evaluated were daunorubicin (DNR), idarubicin, mitoxantrone, semi-synthetic HHT (Stragen Pharma, Geneva, Switzerland) and Mylotarg (Wyeth-Lederle, USA). Mylotarg was not be able to study in K562 and their variant cell lines, because those cell lines are CD33^-^. The IC_50 _of those agents on K562 and HL60 variant cell lines were assessed by MTT assay. The efficacy of P-gp modulation was described by a resistance modifying factor (RMF: ratio of IC_50 _in the presence of zosuquidar or CsA over the IC_50 _in the absence of the P-gp inhibitor). As shown in Table 1, zosuquidar had a greater impact than CsA on drug sensitivity in K562/DOX and HL60/DNR, the two cell lines which express the greatest P-gp activity. For example, 0.3 μM of zosuquidar enhanced the cytotoxicity of DNR in K562/DOX cells more than 45.5-fold; the DNR IC50 decreased from more than 50 μM to 1.1 ± 0.4 μM in the presence of zosuquidar while 2 μM of CsA enhanced the cytotoxicity of DNR in K562/DOX cells only more than 4.8-fold, with the DNR IC50 decreasing to 10.5 ± 1.6 μM in the presence of CsA.

**Table 1:** Modulation of cytotoxicity of K562, HL60 and their variant resistant cells

	**DNR**		**Idarubicin**		**Mitox**		**HHT**		**Mylotarg**	
	**IC50 (μM)**	**RMF**	**IC50 (μM)**	**RMF**	**IC50 (μM)**	**RMF**	**IC50 (ng/ml)**	**RMF**	**IC50 (ng/ml)**	**RMF**

*K562*										
None	0.28 ± 0.09		0.071 ± 0.026		0.60 ± 0.21		57.3 ± 6.5			
Zosuquidar	0.26 ± 0.09	1.1	0.071 ± 0.042	1.0	0.095 ± 0.029	6.3	58.4 ± 10.1	1.0		
CsA	0.23 ± 0.12	1.2	0.068 ± 0.039	1.0	0.14 ± 0.05	4.3	56.6 ± 4.2	1.0		
*K562/HHT40*										
None	2.3 ± 0.2		0.27 ± 0.06		1.7 ± 0.2		197.6 ± 44.4			
Zosuquidar	0.66 ± 0.08	3.5	0.20 ± 0.07	1.4	0.33 ± 0.06	5.2	85.4 ± 2.9	2.3		
CsA	0.51 ± 0.08	4.5	0.24 ± 0.11	1.1	0.45 ± 0.06	3.8	91.3 ± 9.3	2.2		
*K562/HHT90*										
None	3.6 ± 1.5		0.17 ± 0.11		1.9 ± 0.9		409.6 ± 70.2			
Zosuquidar	0.37 ± 0.14	9.7	0.12 ± 0.09	1.4	0.13 ± 0.05	14.6	80.4 ± 8.3	5.8		
CsA	0.32 ± 0.19	11.3	0.10 ± 0.06	1.7	0.25 ± 0.18	7.6	81.8 ± 8.3	5.0		
*K562/DOX*										
None	>50^b^		1.82 ± 0.17		>50		>720			
Zosuquidar	1.1 ± 0.4	>45.5	0.21 ± 0.03	8.7	0.53 ± 0.10	>94.3	88.8 ± 1.9	>8.1		
CsA	10.5 ± 1.6	>4.8	0.30 ± 0.10	6.1	4.29 ± 0.22	>11.7	639.1 ± 70.1	>1.1		
*HL60*										
None	0.16 ± 0.03		0.053 ± 0.004		0.48 ± 0.22		40.5 ± 5.2		131.9 ± 72.3	
Zosuquidar	0.13 ± 0.04	1.2	0.057 ± 0.008	0.9	0.09 ± 0.03	5.3	41.7 ± 3.1	1.0	83.8 ± 9.5	1.6
CsA	0.10 ± 0.02	1.6	0.033 ± 0.008	1.6	0.13 ± 0.07	3.7	33.6 ± 2.6	1.2	35.5 ± 1.0	3.7
*HL60/ADR*										
None	2.1 ± 0.4		0.51 ± 0.03		1.5 ± 0.2		60.7 ± 12.4		>2000	
Zosuquidar	2.4 ± 0.5	0.9	0.50 ± 0.03	1.0	1.4 ± 0.4	1.1	58.6 ± 4.6	1.0	>2000	ND
CsA	0.49 ± 0.04	4.3	0.30 ± 0.09	1.7	0.51 ± 0.03	2.9	48.7 ± 5.3	1.2	67.6 ± 7.6	>29.9
*HL60/DNR*										
None	>40		0.51 ± 0.05		>20		489.9 ± 24.5		>2000	
Zosuquidar	0.49 ± 0.16	>81.6	0.14 ± 0.01	3.6	0.22 ± 0.06	>90.1	46.5 ± 9.7	10.5	620.5 ± 38.9	3.2
CsA	2.5 ± 0.8	>16	0.15 ± 0.00	3.4	1.4 ± 0.6	>14.3	60.3 ± 5.0	8.1	>2000	ND

a. All values are the means determined in quadruplicate in three independent experiments.

b. The maximal dose tested was 50 μM. IC50 was more than 50 μM, not determined.

c. Zosuquidar = 0.3 μM, CsA = 2 μM.

Zosuquidar enhanced the cytotoxicity of DNR in P-gp active cell lines (K562/HHT40, K562/HHT90, K562/DOX and HL60/DNR) but not the MRP active cell line, HL60/ADR. These data indicate that zosuquidar selectively modulates P-gp-mediated resistance. Curiously, zosuquidar enhanced the cytotoxicity of mitoxantrone in two parental cells, K562 and HL60. The viability of these cells with zosuquidar alone was verified to demonstrate that the enhanced cytotoxicity of mitoxantrone in K562 and HL60 cells is not due to toxicity from zosuquidar itself. The reason for this response to Mitox in K562 and HL60 cells is unknown.

### Lack of effect of zosuquidar on wild type BCRP-expressing cells

Daunorubicin and idarubicin are transported by mutant BCRP (R482T or R482G) and not by wild type BCRP (R482), while mitoxantrone is transported by all BCRP variants [20]. It has been shown that zosuquidar did not affect on the mutant BCRP (R482T) mediated drug transport [21]. To assess the effect of zosuquidar on wild type BCRP-mediated drug transport, we evaluated the accumulation of mitoxantrone in K562/BCRP cells that were transfected with wild type BCRP compared to parental K562S cells. Mitoxantrone accumulation in K562/BCRP cells with Zosuquidar treatment was compared to the BCRP specific modulator FTC. 10 μM of FTC enhanced the uptake of mitoxantrone in K562/BCRP cells, while zosuquidar had no effect (D = 0.59 ± 0.11 vs D = 0.04 ± 0.07) (Table 2).

**Table 2:** Effect of zosuquidar on mitoxantrone accumulation in K562/BCRP and K562/Vec cells

	**FTC (10 μM)**	**Zosuquida**
K562/Vec	0	0
K562/BCRP	0.59 ± 0.11	0.04 ± 0.07

### Modulation of drug resistance by zosuquidar in AML patient cells

To determine whether zosuquidar could enhance the chemotherapeutic drug cytotoxicity in primary AML blasts, the effects of zosuquidar on the cytotoxicity of daunorubicin, idarubicin, mitoxantrone, and Mylotarg were examined in 31 AML patient cells. The characteristics of 31 patients are shown in Table 3. In parallel, we analyzed cellular uptake of DIOC_2_(3) in these samples. The effects of CsA on cytotoxicity could not be compared to zosuquidar because CsA alone caused death of 40% to 60% of the primary AML blasts after 48 hour culture. The results in Table 4 present in detail the modulation of cytotoxicity by zosuquidar and the P-gp activity measured by the uptake of DIOC2(3) in those 31 patient cells. Zosuquidar enhanced drug cytotoxicity in 8 of 31 AML cases (26%); among these 8 cases, five cases demonstrated significant P-gp activity (D > 0.3) as determined by cellular uptake of DIOC_2_(3). Among the 31 AML samples evaluated, significant P-gp activity was found in 7 of 31 (23%); zosuquidar enhanced drug cytotoxicity in 5 of these 7 (71%), all of which were older patients > 60 years of age. 24 of 31 AML samples showed no P-gp activity; in 21 of these 24 samples, zosuquidar had no effect on cytotoxicity when combined with chemotherapy. When the samples were divided into two groups based on the ability of zosuquidar to enhance chemotherapeutic cytotoxicity (8 samples with increased cytotoxicity, 23 samples without modification of cytotoxicity), DIOC_2_(3) uptake was significantly different between the two groups (p < 0.004 by Student's t-test) (Table 5).

**Table 3:** Characteristics of patients

Age Mean ± SD (range)	60 ± 17 y (22–81)
Leukocyte (10^9^/L)	46 ± 44 (3.2–184)
FAB subtypes	
1	23% (7/31)
2	23% (7/31)
4	16% (5/31)
5	13% (4/31)
Myelodysplastic syndrome	26% (8/31)
Karyotype	
Favorable	6% (2/31)
Intermediate	71% (22/31)
Poor	19% (6/31)
Not done	3% (1/31)
CD34	
Positive	55% (17/31)
Negative	42% (13/31)
Not done	3% (1/31)
P-gp activity	
Mean D ± SD (range)	0.1 ± 0.1 (0–0.7)
Positive D > 0.3	23% (7/31)
Negative D < 0.3	77% (24/31)

**Table 4:** Modulation of cytotoxicity by zosuquidar and the P-gp activity in 31 patient cells

		**RMF**			**Uptake**
	**Resistance Modifiding**	**DNR/Ida**	**MITOX**	**Mylotarg**	**DIOC2 +Zosu**

P01	No	1.1	0.6	1.0	0.06
P02	Yes/DNR, Ida, Mylotarg	2.3/3.2	20%	35%^e^	0.53
P03	No	0.7	ND	0.8	0.05
P04	No	0.8	ND	1.3	0.1
P05	No	1.1	0.92	1.1	0.05
P06	Yes/Mylotarg	ND	ND	4.1	0.7
P07	Yes/Mylotarg	1.6	ND	29%	0.46
P08	No	1.0	ND	1.2	0.13
P09	No	0.7/1.2	ND	1.0	0.24
P10	No	1.0/1.0	ND	1.0	0.33
P11	No	0.9	ND	13%	0.4
P12	Yes/MITOX	ND	2.1	ND	0.6
P13	No	0.9	1.1	0.9	0.13
P14	No	1.1	ND	ND	0.23
P15	No	1.1	1.3	1.0	0.13
P16	Yes/DNR, Mitox	6.6	2.1	ND	0.04
P17	No	1.0	1.0	0.9	0.05
P18	No	1.1	0.9	1.0	0.06
P19	No	1.0	1.0	1.0	0
P20	Yes/DNR, Mylotarg	1.4	ND	3	0.38
P21	No	ND	ND	0.5	0.12
P22	No	1.2	1	1	0.21
P23	No	1.1	1.0	1.0	0.21
P24	No	ND	ND	1.1	0.06
P25	Yes/DNR, Mitox, Mylo	2.5	2.3	30%	0.05
P26	No	1	ND	ND	0.04
P27	No	0.87	0.82	ND	0.24
P28	No	0.7	1.2	ND	0
P29	Yes/Mylotarg	ND	ND	2.0	0.04
P30	No	1.0	1.1	1.0	0.08
P31	No	1.2	ND	9%	0.26

a. RMF: ratio of IC50 in the presence of zosuquidar over the IC50 in the absence of zosuquidar.

b. The concentration of DNR, Ida and Mitox was varied from 5 to 1000 nM.

c. The concentration of Mylotarg was varied from 12.5 to 1000 ng/ml.

d. ND: Non determiner due to missing the cells, or IC50 was out of the concentration measured.

e. Results on percentage when IC50 could not be able to determine, and X% cell death more in presence of zosuquidar than without zosuquidar.

**Table 5:** Effect of zosuquidar to the cytotoxicity of DNR, Mitox and Mylotarg in 31 AML patient cells

	Modulation*	**No modulation**
Active P-gp (D > 0.3)	71% (5/7)	29% (2/7)
No active P-gp (D < 0.3)	13% (3/24)	87% (21/24)

Modulation* means the enhanced cytotoxicity with zosuquidar.

## Discussion

Multidrug resistance can also result from the overexpression of other member of the ABC transporter family such as MRP1 and BCRP. Overexpression of MRP1 can confer resistance to DNR, doxorubicin, idarubicin, Mitox, HHT and Mylotarg. As for BCRP, its overexpression can also confer resistance to DNR, doxorubicin, idarubicin, Mitox. To verify whether these cells are cross-resistant to those agents, we have well characterized Pgp, MRP1, MRP3 and BCRP expression and activity, which have been shown involving in the chemoresistance of AML treatment [4,22], in K562, HL60 and their derivation cell lines. Non cross-resistance was observed in these cells. In addition, K562 and its derivation cell lines having escalated P-gp activity provide us a good model for this study. These *in vitro *investigations demonstrate that zosuquidar is a highly potent modulator of P-gp-mediated drug resistance. Zosuquidar (0.3 μM) completely or partially restored sensitivity to daunorubicin, idarubicin, mitoxantrone, HHT, and Mylotarg in K562/HHT40, K562/HHT90, K562/DOX and HL60/DNR cells, all of which express P-gp. Zosuquidar was more effective than CsA in enhancing cytotoxicity in the two cell lines with the highest P-gp activity, K562/DOX and HL60/DNR. As it was already described, IC50 of idarubicin was poorly modified for cell lines by P-gp inhibitors. In the other hand, P-gp efflux in clinical samples had a negative impact on complete remission achievement in patients treated with idarubicin [23].

It has been shown that zosuquidar did not affect on the mutant BCRP (R482T) mediated drug transport [21]. In this paper we have shown that Zosuquidar does not affect on the wild type BCRP mediated drug transport, it selectively modulates P-gp-mediated resistance, but has no effect on MRP or wild type BCRP-mediated resistance.

Zosuquidar enhanced the cytotoxicity of mitoxantrone in two parental cells, K562 and HL60. The reason for this response to Mitox in K562 and HL60 cells is unknown. It is possible that other non identified ABC proteins confer Mitox resistance in these two cell lines. It needs the further investigations.

The *in vitro *studies of primary AML blasts demonstrate that zosuquidar enhanced the cytotoxicity of daunorubicin, idarubicin, mitoxantrone, or Mylotarg in the majority of AML cases which expressed active P-gp. These results suggest that zosuquidar may have great potential as a chemosensitizing agent in combination with a number of standard agents for AML patients whose blasts express P-gp. Several clinical trials have given the encouraging results [14,15,24]. The clinical phase I trial of zosuquidar, co-administered with daunorubicin and cytosine arabinoside (ARA-C) on sixteen patients with AML [15] has shown that eleven patients achieved a complete remission and one a partial remission with a median survival of 559 (range 38–906) days. Non-hematologic grade 3 and 4 toxicities were seen in 4 patients. Zosuquidar infusion was associated with rapid inhibition of Rh123 efflux in CD56^+ ^cells in 16/16 patients and in CD33^+ ^cells in 6/10 patients. Other clinical trials in patients with non-Hodgkin's lymphoma [24] and patients with advanced malignancy [14] have shown also that zosuquidar did not significantly affect the pharmacokinetics of doxorubicin and had moderate effects on the pharmacokinetics of vincristine. These clinical trials approved that zosuquidar could safely administrated with daunorubicin, doxorubicin and other regimens. Zosuquidar is potent, specific, and avoids the pharmacokinetic interactions that limit the use of other P-gp inhibitors. P-gp expression is particularly frequent in AML patients older than age 60 years, a subgroup of patients with poor induction response and long-term outcomes. This patient subgroup may particularly benefit from treatment strategies combining chemotherapy with zosuquidar and phase II trials are currently ongoing.

## Conclusion

Zosuquidar completely or partially restored drug sensitivity in all P-gp-expressing leukemia cell lines tested and enhanced the cytotoxicity of anthracyclines (daunorubicin, idarubicin, mitoxantrone) and gemtuzumab ozogamicin (Mylotarg) in primary AML blasts with active P-gp. In addition, the cytotoxicity enhanced by Zosuquidar was found to be more potent than that by cyclosporine A in cells with highly active P-gp. These in vitro studies suggest that zosuquidar may be an effective adjunct to cytotoxic chemotherapy for AML patients whose blasts express P-gp, especially for older patients than age 60 years.

## Competing interests

The author(s) declare that they have no competing interests.

## Authors' contributions

RT performed the major experiments, its design and manuscript. AMF and JYP participated all flow cytometry analysis. ZM collected AML patient blood. SC and TS assisted blood cell and drug preparation. HM helped us for BCRP study. OL and JPM participated in its design and the manuscript revision. All authors read and approved the final manuscript.

## Pre-publication history

The pre-publication history for this paper can be accessed here:


